# *Polygonum multiflorum* Thunb.: A Review on Chemical Analysis, Processing Mechanism, Quality Evaluation, and Hepatotoxicity

**DOI:** 10.3389/fphar.2018.00364

**Published:** 2018-04-16

**Authors:** Yue Liu, Qi Wang, Jianbo Yang, Xiaohan Guo, Wenxi Liu, Shuangcheng Ma, Shaoping Li

**Affiliations:** ^1^School of Traditional Chinese Medicine, Beijing University of Chinese Medicine, Beijing, China; ^2^Institute for Control of Chinese Traditional Medicine and Ethnic Medicine, National Institutes for Food and Drug Control, Beijing, China; ^3^School of Chinese Materia Medica, Beijing University of Chinese Medicine, Beijing, China; ^4^State Key Laboratory for Quality Research in Chinese Medicine, University of Macau, Macao, China

**Keywords:** *Polygonum multiflorum* thunb., chemical analysis, processing mechanism, quality evaluation, hepatotoxicity, stilbene glucosides, anthraquinones, review

## Abstract

*Polygonum multiflorum* Thunb. and its processed products have been used in China for centuries due to their multiple beneficial effects to human body. Currently, liver injuries caused by taking *P. multiflorum* have been reported worldwide, but the potential toxic components and possible mechanism that caused hepatotoxicity remain unclear. It is worth noting that the processing procedure could significantly decrease the toxicity of raw *P. multiflorum* and the processed products of *P. multiflorum* are considered to be relatively safe. However, the processing mechanism is still ambiguous, and there is the lack of a scientific approach to control the quality of *P. multiflorum* praeparata. This study is the first review that summarizes the recently advances (from 2007 to 2017) in the chemical analysis of *P. multiflorum*, and provides comprehensive information on the quantitative and qualitative analysis of *P. multiflorum* as well as its related species. In addition, the processing mechanism and quality evaluation of processed *P. multiflorum* are discussed. Moreover, the toxicity of *P. multiflorum* is analyzed from the perspectives of exploration of the proposed toxic ingredients, metabolite identification, metabolomics studies, and exogenous contaminant determination. Furthermore, trends and perspectives for future research of this medicine are discussed.

## Introduction

According to traditional Chinese medicine (TCM) theory, *Polygonum multiflorum* Thunb. (PM) is one representative drug that possesses different efficacies in its crude and processed forms (Supplementary Figure S1). *P. multiflorum* praeparata (PMP) is used more frequently in clinical practice mainly because of its tonic and anti-aging effects, whereas PM is commonly applied to resolve toxins, moisten the intestines and free stools (China Pharmacopoeia Committee, 2015). Modern pharmacological studies and clinical practice have indicated these two medicines have various biological activities, including anti-tumor, anti-oxidative, anti-bacterial, anti-hyperlipidemia, anti-atherosclerosis, immunomodulating and hepatoprotective effects (Bounda and Feng, 2015; Lin et al., 2015a; Li et al., 2016a). The chemical profiles demonstrate that stilbenes and anthraquinones are the major characteristic constituents, of which 2,3,5,4′-tetrahydroxystilbene-2-*O*-β-glucoside (TSG), emodin-8-*O*-β-D-glucoside (EMG), and physcion-8-*O*-β-D-glucoside (PG) are found to be dominant in PM, while PMP mainly contains TSG, emodin and physcion (Bounda and Feng, 2015; Lin et al., 2015a; Li et al., 2016a). These compounds are widely believed to be responsible for the bioactivities of PM and PMP. Studies have shown that TSG exhibited many medicinal properties, including delaying the senescence effect, cardiovascular protection, neuroprotective effects, and the promotion of hair growth (Ling and Xu, 2016). On the other hand, anthraquinones also possessed a wide spectrum of pharmacological properties, such as anti-cancer, anti-microbial, anti-inflammatory, anti-oxidant and hepatoprotective activities (Zhou et al., 2015; Dong et al., 2016; Sun et al., 2016). The previous reviews focusing on the botany, phytochemistry, pharmacological effects, toxicology and some other different aspects of PM are listed in Table 1 (Zhang et al., 2009; Shaw, 2010; Sun and Zhang, 2010; Teschke et al., 2014a,b, 2015b, 2016; Wang et al., 2014; Bounda and Feng, 2015; Lee et al., 2015; Lei et al., 2015; Lin et al., 2015a; Teschke and Eickhoff, 2015a; Zhou et al., 2015; Dong et al., 2016; Li et al., 2016a; Ling and Xu, 2016; Sun et al., 2016; Zhang P. et al., 2016).

**Table 1:** Overview of PM-related reviews since 2007.

**Topic**	**References**
Traditional usages and botany	Bounda and Feng, 2015; Lin et al., 2015a
Phytochemistry/Bioactive compounds	Bounda and Feng, 2015; Lin et al., 2015a
Pharmacology	Bounda and Feng, 2015; Lin et al., 2015a; Zhou et al., 2015; Dong et al., 2016; Li et al., 2016a; Ling and Xu, 2016; Sun et al., 2016
Clinical studies/Hepatotoxicity case reports	Zhang et al., 2009; Sun and Zhang, 2010; Teschke et al., 2014a,b, 2015b, 2016; Wang et al., 2014; Bounda and Feng, 2015; Lee et al., 2015; Lei et al., 2015; Teschke and Eickhoff, 2015a; Zhang P. et al., 2016
Side effect and safety	Shaw, 2010; Bounda and Feng, 2015; Lin et al., 2015a; Dong et al., 2016; Li et al., 2016a
Pharmacokinetics	Bounda and Feng, 2015; Lin et al., 2015a; Dong et al., 2016
TSG	Ling and Xu, 2016
Emodin	Dong et al., 2016
Rhein	Zhou et al., 2015; Sun et al., 2016

Nowadays, there are two big problems that seriously hamper the research and development of PM. First, increasing cases related to the hepiatic lesions induced by PM have been reported in China and other countries (But et al., 1996; Park et al., 2001; Mazzanti et al., 2004; Panis et al., 2005; Shaw, 2010; Sun and Zhang, 2010; Jung et al., 2011; Dong et al., 2014; Teschke et al., 2014b; Wang et al., 2014; Lee et al., 2015; Lei et al., 2015; Zhang P. et al., 2016), which draw great attention from scholars. It is worth noting that the initially references were published in Europe. From 2004 to 2010, 10 cases of adverse reactions associated with the intake of Shou-Wu-Pian (is the tablet form of the root tuber of PM) were reported in Italy (Mazzanti et al., 2004; Valente et al., 2010), the Netherlands (Panis et al., 2005), and England (Zhang et al., 2009; Furukawa et al., 2010; Teschke et al., 2014a,b, 2015b, 2016; Stickel and Shouval, 2015; Teschke and Eickhoff, 2015a), respectively. Among these cases, Shou-Wu-Pian was commonly used for hair care, besides one case for the treatment of chronic prostatitis. The age of patients ranged from 5 to 78 years, and 7 of them were female. The duration with ingestion of Shou-Wu-Pian lead to the liver injury ranged from 2 weeks to several months. In the end, all these 10 patients recovered from symptoms of hepatic dysfunction after they discontinued the consumption of Shou-Wu-Pian. With regard to the hepatotoxicity of PM, Shaw (2010) proposed that the incorrect use of PM might be the leading cause, which mainly due to the patients believed herbal medicines were the natural products and harmless, as well as they usually used these preparations without medical supervision. In another hand, anthraquinones and contaminants (mycotoxins, heavy metals, and pesticides) were considered to be the main hepatotoxic components (Ernst, 2002; Mazzanti et al., 2004; Panis et al., 2005; Furukawa et al., 2010), but this issue was still in dispute due to the lack of convincing evidence. In recent years, extensive experiments have been performed both *in vivo* and *in vitro*, unfortunately, the potential toxic components and possible mechanism that caused the hepatotoxicity remain unclear (Lin et al., 2015a; Wang J. et al., 2015; Li et al., 2016a; Wang et al., 2016). Second, processing is a very important procedure that played significant roles in the toxicity-attenuating effect as well as the enhancing tonifying efficacy (Lin et al., 2015a; Wang J. et al., 2015; Li et al., 2016a; Wang et al., 2016; Cui et al., 2017), and the quality assurance of PMP is believed to be the foundation of its clinical usage. Nevertheless, the processing mechanism is still ambiguous, and the current pharmacopeia protocols (China Pharmacopoeia Committee, 2015) failed to differentiate PM from PMP mainly due to their poor specificity (Table 2), and as far as we know, there is no appropriate method to evaluate whether PMP is completely processed or not. These two independent and closely implicated questions are the hot spots of PM research in the future.

**Table 2:** Quality standards recorded in Chinese pharmacopeia.

**Samples**	**Analytes**	**HPLC assay**	**UV**	**Limitation**
PM	TSG	Eluted with acetonitrile: water (25: 75)	320 nm	TSG not <1.0%
	Emodin and Physcion^a^	Eluted with methanol: 0.1% formic acid aqueous solution (80: 20)	254 nm	Combined anthraquiones not <0.10%
PMP	TSG	Eluted with acetonitrile: water (25: 75)	320 nm	TSG not <0.70%
	Emodin and Physcion^b^	Eluted with methanol: 0.1% formic acid aqueous solution (80: 20)	254 nm	Free anthraquinones not <0.10%
*P. multiflorum* caulis	TSG	Eluted with acetonitrile: water (26: 74)	320 nm	TSG not <0.20%

a *indirect quantification*.

b *direct quantification*.

This study reviews the recently advances in the chemical analysis of PM and PMP (from 2007 to 2017), which provides comprehensive information on the quantitative and qualitative analysis of PM and its related species. In addition, the processing mechanism and quality evaluation of PMP are discussed. Moreover, the toxicity of PM is analyzed from the perspectives of proposed toxic ingredient exploration, metabolite identification, metabolomics studies and exogenous contaminant determination. In addition, trends and perspectives for future research of this TCM are discussed. The Schematic diagram of the review process is shown in Supplementary Figure S2.

## Chemical constituents and quality markers

**Figure 1:**
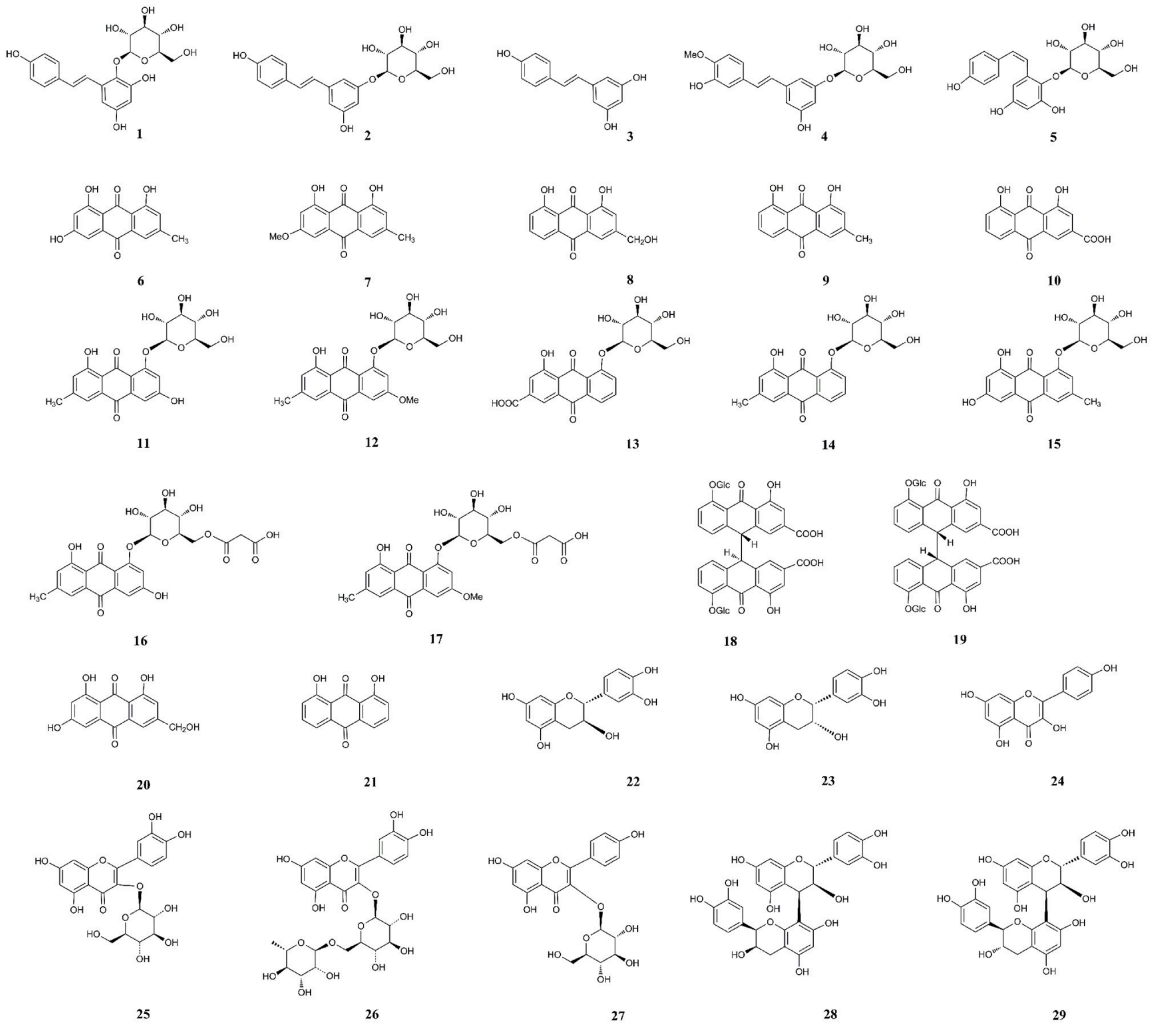
Chemical structures of stilbenes, anthraquinones and flavonoids.

**Figure 2:**
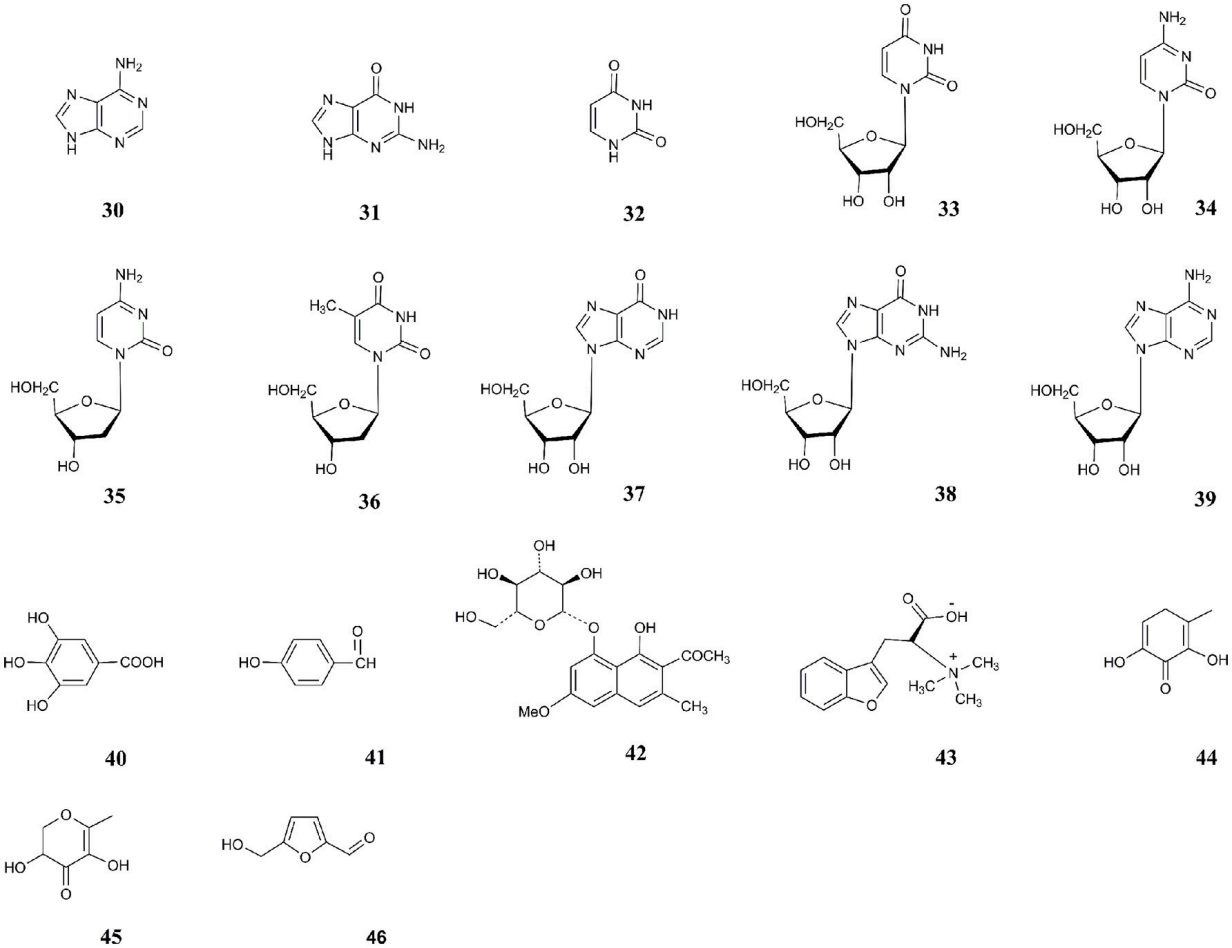
Chemical structures of nucleosides and other compounds.

The chemical constituents and pharmacological activities of PM were reviewed in last 2 years (Bounda and Feng, 2015; Lin et al., 2015a), and more than 103 constituents have been isolated and identified, which included stilbenes, quinones, flavonoids, phospholipids, and other compounds. Among these ingredients, stilbene glucosides and anthraquinones are recognized as two major characteristic constituents of PM. Forty six biologically active components or quality markers mentioned in the publications that focused on the topic of “Chemical analysis” of PM were summarized. The chemicals are described as follows (Figures 1, 2): stilbenes: TSG (1), polydatin (2), resveratrol (3), rhaponiticin (4), and *cis*-TSG (5); anthraquinones: emodin (6), physcion (7), aloe-emodin (8), chrysophanol (9), rhein (10), EMG (11), PG (12), rhein-8-*O*-β-D-glucoside (RHG) (13), chrysophanol-8-*O*-β-D-glucoside (CHG) (14), emodin-1-*O*-β-D-glucoside (EMG1) (15), emodin-8-(6′-*O*-malonyl)-glucoside (16), physcion-8-(6′-*O*-malonyl)-glucoside (17), sennoside A (18), sennoside B (19), 6-OH-emodin (20) and danthron (21); flavonoids: catechin (22), epicatechin (23), quercetin (24), hyperin (25), rutin (26), astragalin (27), proanthocyanidin B1 (28), and proanthocyanidin B2 (29); nucleosides: adenine (30), guanine (31), uracil (32), uridine (33), cytidine (34), 2′-deoxycytidine (35), thymidine (36), inosine (37), guanosine (38), and adenosine (39); and phenolic acids and other compounds: gallic acid (40), *p*-hydraxy benzaldehyde (41), troachrysone-8-*O*-β-D-glucoside (TOG) (42), hypaphorine (43), hydroxymaltol (44), 2,3-dihydro-3,5-dihydroxy-6-methyl-4(H)- pyran-4-one (DDMP) (45), and 5-hydroxymethylfurfural (5-HMF) (46).

## Chemical analysis of PM

### Qualitative determination

During the past decade, mass spectrometry (MS) and its combination with chromatographic separation techniques have emerged as crucial approaches to describe the chemical profiles of PM, which including high performance liquid chromatography-ion trap-mass spectrometry (HPLC-IT-MS) (Sun et al., 2009a), high performance liquid chromatography-electrospray ionization-mass spectrometry (HPLC-ESI-MS) (Yi et al., 2007; Zhao et al., 2013), ultra performance liquid chromatography-quadrupole-time of flight-mass spectrometry (UPLC-Q-TOF-MS) (Lin et al., 2015c; Wang et al., 2017), and high-performance liquid chromatography/ultra high pressure liquid chromatography-linear ion trap-Orbitrap hybrid mass spectrometry (HPLC/UHPLC-LTQ-Orbitrap-MS) (Xu et al., 2012; Qiu et al., 2013; Wang T. H. et al., 2015), and these results suggest that more than 135 compounds were detected and identified by comparison with the standards or through investigating references. It should be noted that 28 new dianthrone glycosides were characterized for the first time by the means of HPLC-LTQ-Orbitrap-MS (Xu et al., 2012), and their fragmentation behaviors were also proposed. This finding enriched the chemical structure types of PM and offered us more comprehensive information. Actually, several novel dianthrone glycosides from PM were elucidated by the conventional phytochemistry methods 4 years later (Yang J. B. et al., 2016; Yang et al., 2017a,b), which confirmed the existence of dianthrone glycosides in PM. Compared with traditional Q-TOF mass spectrometry, UPLC-LTQ-Orbitrap-MS^n^ provides superior resolution and mass accuracy; meanwhile, with the MS^n^ function, the Orbitrap technology can achieve 10 levels of MS analysis capability (Lin et al., 2015d). In this manner, the phenolic constituents were rapidly screened in the roots of PM, and based on the fragment pattern rules of reference stilbenes and anthraquinone derivatives, 59 constituents were characterized or tentatively identified, of which 22 constituents were the first to be reported in PM and 12 compounds were characterized as potential new compounds (Qiu et al., 2013). Table 3 summarizes the chromatographic approaches for the qualitative analysis of PM.

**Table 3:** Chromatographic approaches for the qualitative analysis of PM.

**Techniques**	**Analytes**	**Samples**	**Details**	**References**
HPLC-IT-MS	12 glycosides including 3 newly reported	Crude root	Eluted with CH_3_OH: 10 mM CH_3_COONH_4_ (0 min: 0:100; 20 min: 30:70; 50 min: 95:5; 60 min: 95:5) on an Ultimate XB-C_18_ column	Sun et al., 2009a
HPLC-ESI-MS	11 compounds including 2 unknowns	Crude root	Eluted with H_2_O: CH_3_CN (both of them containing 0.5% CH_3_COOH) (0 min: 90:10; 35 min: 60:40; 50 min: 0:100) on an Alltima C_18_ column	Yi et al., 2007
HPLC-ESI-MS	7 compounds	Caulis	Eluted with CH_3_CN: H_2_O (containing 0.5% HCOOH) (0 min: 0:100; 22 min: 16:84; 45 min: 34:66; 60 min: 38:62; 70 min: 95:5; 80 min: 95:5) on a Grace Alltima C_18_ column	Zhao et al., 2013
UPLC-Q-TOF-MS	29 components including 8 newly reported	Crude root	Eluted with CH_3_OH: H_2_O (both of them containing 0.1% CH_3_COOH) (0 min: 0:100; 25 min: 35:65; 40 min: 70:30; 50 min: 100:0; 53 min: 100:0; 53.1 min: 0:100; 60 min: 0:100) on a Phenomenex Hydro-RP C_18_ column	Lin et al., 2015c
UHPLC-Q-TOF-MS	131 compounds including 26 unknowns	Crude root	Eluted with CH_3_CN: H_2_O (containing 0.1% CH_3_COOH) (0 min: 97:3; 20 min: 3:97) on a T3 C_18_ column	Wang et al., 2017
HPLC-LTQ-Orbitrap-MS	28 new dianthrone glycosides	Crude root	Eluted with CH_3_CN: H_2_O (containing 0.1% HCOOH) (0 min: 5:95; 6 min: 15:85; 12 min: 15:85; 25 min: 38:62; 30 min: 70:30) on a Hypersil Gold C_18_ column	Xu et al., 2012
UHPLC-LTQ-Orbitrap-MS	59 phenolic compounds including 12 newly reported	Crude root	Eluted with CH_3_CN: H_2_O (containing 0.1% HCOOH) (0 min: 5:95; 6 min: 15:85; 12 min: 15:85; 25 min: 38:62; 30 min: 70:30; 35 min: 90:10) on a Hypersil Gold C_18_ column	Qiu et al., 2013
UHPLC-LTQ-Orbitrap-MS	25 compounds	Crude and processed root	Eluted with CH_3_CN: H_2_O (containing 0.1% HCOOH) (0 min: 13:87; 3.5 min: 35:65; 7.5 min: 90:10; 8.5 min: 95:5; 10 min: 95:5) on an AcQuity UPLC™ BEH C_18_ column	Wang T. H. et al., 2015

### Quantitative analysis

Stilbenes are one of the major components in PM. To date, more than 20 stilbenes and analogs have been found, of which TSG is the most representative compound. Previous publications have demonstrated that TSG possessed anti-tumor, anti-aging and liver-protective bioactivities (Bounda and Feng, 2015; Lin et al., 2015a; Ling and Xu, 2016), which matched well with the traditional efficacies of PM. Quinones are the other characteristic components in PM, which had anti-microbial, anti-cancer, anti-oxidant and anti-human cytomegalovirus effects (Bounda and Feng, 2015; Lin et al., 2015a; Zhou et al., 2015; Dong et al., 2016; Sun et al., 2016). Due to the long conjugated system existing in basic structures of stilbenes and anthraquinones, their characteristic ultraviolet absorptions are easily screened. Therefore, HPLC in tandem with an ultraviolet detector (UV) or diode array detector (DAD)/photodiode array detector (PDA) are widely applied in the quantitative evaluation of PM (Yi et al., 2007; Han et al., 2009, 2013; Jiao and Zuo, 2009; Yan et al., 2010; Zhao et al., 2013; Liang et al., 2014; Li et al., 2016b) According to the Chinese pharmacopeia, TSG, emodin and physcion were eluted on a C_18_ column by the means of HPLC. However, due to the lack of standard references of anthraquinone glycosides, the content of combined anthraquinone was determined by an indirect method, an additional acidic hydrolysis step was needed, and then, the resulting aglycones were assessed. In this way, combined anthraquinones in PM were calculated as the total amount of physcion and emodin. In fact, determination of the authentic composition has always played a vital role the in quality control of herbals, and with the increasing availability of anthraquinone glycoside standards, direct quantification of the combined anthraquinones was performed by some researchers. Their contents of five markers, i.e., TSG, EMG, PG, emodin and physcion, were quantitatively evaluated by HPLC with DAD (Yi et al., 2007). The results indicated that TSG and EMG were the predominant compounds in PM, which account for about 2.6~4.2% and 0.2~0.6%, respectively, of the total dry weight, and the contents of the other three constituents were no more than 0.06%. Another HPLC-DAD approach (Han et al., 2013) was also proposed for the simultaneous determination of 8 hydrophilic bioactive compounds of PM including TSG, EMG, gallic acid, catechin, epicatechin, hypaphorine, and proanthocyanidin B1 as well as B2, which results in similar data. On the other hand, when analytes are present in trace amounts or showed poor separation, combined MS and liquid chromatographic techniques are the preferred alternative, which provided higher sensitivity and selectivity. ESI-MS in the negative mode was most commonly used in the quantitative analysis of PM (Liang et al., 2011; Zhu et al., 2012; Lin et al., 2015b; Wang T. H. et al., 2015; Luo et al., 2016a). An HPLC-MS/MS method was developed for the simultaneous determination of 14 compounds including stilbenes, quinones, flavonoids and phenolic acids, which might be the work that quantified most compounds in PM. Apart from approaches utilizing HPLC in tandem with UV, DAD, and MS, capillary gas chromatography coupled with flame ionization and mass spectrometric detection (GC-FID-MS) (Zuo et al., 2008) and micellar electrokinetic chromatography (MECK) (Lao et al., 2013; Luo et al., 2015a,b, 2016b) were also established for the determination of stilbenes and anthraquinones in PM. However, due to the complicated protocols and additional derivatization step, GC and MECK might not be substitutes for HPLC as a routine test method. Table 4 summarizes the chromatographic methods for the quantitative analysis of PM.

**Table 4:** Quantitative analysis methods of PM.

**Techniques**	**Analytes**	**Samples**	**Details**	**References**
HPLC-PDA (290, 320 nm)	TSG, emodin, physcion, EMG, PG	Crude root	Eluted with H_2_O: CH_3_CN (both of them containing 0.5% CH_3_COOH) (0 min: 90:10; 35 min: 60:40; 50 min: 0:100) on an Alltima C_18_ column	Yi et al., 2007
HPLC-PDA (254 nm)	emodin, physcion, aloe-emodin, rhein, chrysophanol	Crude root	Eluted with CH_3_OH: H_2_O: H_3_PO_4_ (0 min: 600:400:1; 80 min: 600:400:1) on an Agilent C_18_ reversed-phase column	Jiao and Zuo, 2009
HPLC-UV (254, 320 nm)	TSG, emodin, physcion	Crude root	Eluted with CH_3_CN: H_2_O (25:75) on a Diamond C_18_ analytical column; Eluted with CH_3_OH: 0.1% H_3_PO_4_ (85:15) on a Diamond C_18_ analytical column	Yan et al., 2010
HPLC-DAD (210, 280, 320 nm)	TSG, EMG, gallic acid, catechin, epicatechin, proanthocyanidin B1 and B2, hypaphorine	Crude root, rhizome, stem	Eluted with CH_3_CN: H_2_O (containing 0.05% H_3_PO_4_) (0 min: 0:100; 7 min: 6:94; 12 min: 6:94; 20 min: 8:92; 22 min: 12:88; 50 min: 25:75) on a Zorbax SB-AQ column	Han et al., 2013
HPLC-UV (254, 320 nm)	TSG, emodin, physcion	Crude root	Eluted with CH_3_OH: H_2_O (containing 0.1% H_3_PO_4_) (30:70) and (80:20) on a Waters Nova-Pak C18 column, respectively	Liang et al., 2014
HPLC-UV (254, 320 nm)	TSG, emodin, physcion	Crude root	Eluted with CH_3_CN: H_2_O (25:75) and CH_3_OH: H_2_O (containing 0.1% H_3_PO_4_) (80:20) on a SinoChrom ODS BP C_18_ RP column, respectively	Li et al., 2016b
UPLC-PDA (280, 320 nm)	TSG, EMG, emodin, physcion	Crude and processed root	Eluted with H_2_O: CH_3_CN (both of them containing 0.3% CH_3_COOH) (0 min: 85:15; 2 min: 85:15; 3 min: 75:25; 5 min: 70:30; 6 min: 15:85; 7 min: 0:100) on an Acquity BEH C_18_ column	Han et al., 2009
HPLC-PDA (290 nm)	TSG, emodin, physcion	Caulis	Eluted with CH_3_CN: H_2_O (containing 0.5% HCOOH) (0 min: 0:100; 22 min: 16:84; 45 min: 34:66; 60 min: 38:62; 70 min: 95:5; 80 min: 95:5) on a Grace Alltima C_18_ column	Zhao et al., 2013
HPLC-MS	TSG, emodin, physcion	Crude root	Eluted with CH_3_CN: H_2_O (both of them containing 0.5% CH_3_COOH) (0 min: 10:90; 45 min: 35:65; 65 min: 100:0) on an Alltima C_18_ analytical column	Liang et al., 2011
HPLC-MS/MS	TSG, emodin, physcion, gallic acid, resveratrol, polydatin, catechin, epicatechin	Crude and processed root	Eluted with CH_3_CN: H_2_O (containing 0.05% HCOOH) (0 min: 10:90; 10 min: 60:40; 15 min: 90:10; 17 min: 10:90; 20 min: 10:90) on an Eclipse Plus C_18_ column	Zhu et al., 2012
HPLC-MS/MS	TSG, emodin, physcion, EMG, RHG, resveratrol, polydatin, catechin, rutin, epicatechin, gallic acid, rhaponiticin, hyperin, p-hydraxy benzaldehyde	Crude root	Eluted with CH_3_OH: H_2_O (both of them containing 0.1% HCOOH) (0 min: 20:80; 2 min: 40:60; 4 min: 50:50; 6 min: 60:40; 8 min: 70:30; 10 min: 80:20; 12 min: 100:0; 15 min: 100:0; 15.1 min: 0:100; 20 min: 20:80) on a Phenomenex Hydro-RP C_18_ column	Lin et al., 2015b
UHPLC-LTQ-Orbitrap-MS	TSG, EMG, emodin, gallic acid	Crude and processed root	Eluted with CH_3_CN: H_2_O (containing 0.1% HCOOH) (0 min: 13:87; 3.5 min: 35:65; 7.5 min: 90:10; 8.5 min: 95:5; 10 min: 95:5) on an AcQuity UPLC™ BEH C_18_ column	Wang T. H. et al., 2015
UPLC-MS/MS	TSG, EMG, aloe-emodin, emodin, rhein, physcion, resveratrol, polydatin, rutin, epicatechin, gallic acid, quercetin, astraglin, hyperoside	Crude root	Eluted with CH_3_CN: H_2_O (containing 0.1% HCOOH) (0 min: 100:0; 1 min: 90:10; 2 min: 10:90; 3 min: 10:90; 4 min: 90:10; 5 min: 90:10) on a Waters BEH C_18_ column	Luo et al., 2016a
Capillary-GC-FID-MS	emodin, physcion, aloe-emodin, rhein, chrysophanol	Crude root	The temperature program was 0 min: 180°C; 1 min: 180°C; 11 min: 300°C; 21min: 300°C on a EC^TM^-5 capillary column	Zuo et al., 2008
MEKC (210 nm)	TSG, proanthocyanidin B1 and B2, gallic acid, catechin, epicatechin, hypaphorine	Crude root	Optimum separation was obtained within 14 min by using 50 mM phosphate buffer containing 90 mM SDS and 2% (m/v) HP-β-CD (pH 2.5) at 15 kV and 20°C	Lao et al., 2013

Besides the stilbenes and quinones, nucleosides and nucleobases also have been determined by MS detection (Luo et al., 2015c,d,e, 2016c,d; Xu et al., 2015).

### Comparative analysis of related medicinal plants

According to the Chinese pharmacopeia, PM, *Polygonum cuspidatum* (PC) and *Rheum officinale* Baill. (RO) are the most frequently used traditional Chinese medicines in the family polygonaceae, which contribute to a wide range of pharmaceutical properties. Due to the similar types of constituents contained in these similar medicinal plants, several studies focused on the quantification and discrimination have been carried out by means of HPLC in tandem with UV or MS (Avula et al., 2007; Huang et al., 2008; Ma et al., 2012; Li et al., 2014; Feng et al., 2016). In HPLC-MS (Huang et al., 2008; Li et al., 2014; Feng et al., 2016), more than 30 compounds had been identified, which mainly belonged to stilbenes, anthraquinones, phenolic acids, and flavonoids. Among these components, thirteen analyte markers including TSG, EMG, emodin, physcion, aloe-emodin, rhein, chrysophanol, piceid, resveratrol, epicatechin, gallic acid, and sennoside A as well as B were simultaneously determined by an HPLC-variable wavelength detection (VWD) approach (Ma et al., 2012). The vital characteristic components for the quality control of a single herb were found by systematic comparison of the chemical compositions of these three herbs, and the results demonstrated that stilbenes and anthraquinones were the main constituents, while chrysophanol, TSG, and piceid could be used as key markers in the discrimination of RO, PM, and PC, respectively. Besides the routine assays mentioned above, HPLC with fluorescence detection (He et al., 2009) and ^1^H-NMR approach (Frederich et al., 2011) were also achieved for the quality assessment of these polygonaceous herbs. In addition, the descriptions of macroscopic and microscopic properties also played significant roles in the authentication of the raw materials and their adulteration (Avula et al., 2007; Liang et al., 2011, 2014). Table 5 summarizes the chromatographic methods for the comparative analysis of PM and its related medicinal plants.

**Table 5:** Comparative analysis methods of PM-related medicinal plants.

**Techniques**	**Analytes**	**Samples**	**Details**	**References**
HPLC-VWD/DAD (254, 280, 320 nm)	TSG, emodin, physcion, aloe-emodin, rhein, chrysophanol, EMG, piceid, resveratrol, epicatechin, gallic acid, sennoside A and B	PM, PC, and RO	Eluted with CH_3_CN: H_2_O (containing 0.05% HCOOH) (0 min: 5:95; 2 min: 10:90; 4 min: 15:85; 10 min: 15:85; 11 min: 21:79; 14 min: 21:79; 21 min: 29:71; 23 min: 40:60; 25 min: 50:50; 26 min: 50:50; 28 min: 80:20; 30 min 100:0; 32 min: 100:0) on an Agilent Zorbax Stable Bond-C_18_ column	Ma et al., 2012
HPLC-PDA (280, 320 nm)	polydatin, resveratrol, aloe-emodin, rhein, emodin, physcion, danthron, chrysophanol	PM, PC, *P. aviculare, P. bristorta*, and *P. vulgare*	Eluted with H_2_O: CH_3_CN (both containing 0.1% CH_3_COOH) (0 min: 80:20; 35 min: 0:100) on a Phenomenex Gemini C_18_ column	Avula et al., 2007
HPLC-DAD-ESI/MS (290 nm)	TSG, rhaponticoside, resveratrol, piceid, aloe-emodin, emodin, physcion, rhein, chrysophanol, EMG, PG, TOG, EMG1, CHG	PM, RO, and *P. reynoutria*	Eluted with H_2_O: CH_3_CN (0 min: 85:15; 10 min: 80:20; 40 min: 47:53; 60 min: 0:100) on an Alltima C_18_ column	Feng et al., 2016
HPLC-DAD-ESI/MS^n^ (290 nm)	TSG, *cis*-TSG, resveratrol, piceid, resveratroloside, emodin, physcion, EMG, PG, EMG1	PM and PC	Eluted with CH_3_CN: H_2_O (containing 0.1% HCOOH) (0 min:22:78; 1.5 min: 30:70; 6 min: 65:35; 8 min: 90:10; 9 min: 90:10) on a Kinetex C_18_ column	Li et al., 2014
HPLC- fluorescence detection (440, 540 nm)	emodin, physcion, rhein, aloe-emodin, chrysophanol	PM and PC	Eluted with CH_3_OH: H_2_O (containing 0.1% HCOOH) (0 min: 85:15; 15 min: 85:15) on a Hypersil C_18_ column	He et al., 2009
NMR	Fingerprint analysis	PM and PC	Performed on a Bruker Avance 500 MHz NMR spectrometer operating at 500.13 MHz	Frederich et al., 2011

## Chemical analysis of PMP

### Investigation of the processing mechanism

On the basis of TCM theory, most of the herbs need to be processed before their clinical usage, and during this procedure, the appearance characteristics and bioactivities of herbs might be changed. Thus, different herbal medicine forms will be selected precisely according to the diagnostics of patients. PM is one typical medicine that has completely different utilities in the crude and processed forms, and especially, the toxicity-attenuating effect of processing has been verified (Yu et al., 2011; Wu et al., 2012; Lin et al., 2015a,e; Wang J. et al., 2015; Li et al., 2016a; Wang et al., 2016; Cui et al., 2017). These fascinating variations have attracted great attention from scientists, and considerable efforts were performed to explore the processing mechanism of PM. Various approaches were applied to monitor the transformation of principal compounds, and the results suggested that the hydrolysis reaction and Maillard reaction were involved in the steaming process of the root of PM (Liu et al., 2009, 2013; Xu et al., 2011; Yu et al., 2011; Chen et al., 2012; Wu et al., 2012; Yang et al., 2015; Zhai et al., 2016; Sun et al., 2017; Zhao et al., 2017). First, the combined anthraquinones were unequivocally hydrolyzed into free ones. Emodin and physcion were the characteristic components in PMP, and the content of emodin was increased by more than 30% after processing, while the content of EMG decreased. Meanwhile, a similar downswing was found regarding the level of TSG, and approximately 60% of TSG was reduced during PMP preparation (Yu et al., 2011; Chen et al., 2012; Wu et al., 2012; Lin et al., 2015e; Yang et al., 2015). It should be noted that when the steaming time was extended, more than 90% of TSG disappeared (Chen et al., 2012). However, the hydrolysis products of TSG could not be detected by most researchers who focused on the rules of PMP processing, and only one publication indicated that a deglycosylated compound had the [TSG+H-Glu+H_2_O]^+^ ion at *m/z* 245.0, which was observed in a direct ionization mass method (Hu et al., 2012). Actually, exploration the products of TSG during the processing is a research project with important scientific significance, which would play a vital role in improving our understanding on the global processing mechanisms of PM. Apart from stilbenes and quinones, the concentration of gallic acid was significantly increased, which might be associated with the hydrolysis of tannin (Chen et al., 2012; Zhai et al., 2016; Zhao et al., 2017). Secondly, the occurrence of a Maillard reaction would likely be responsible for the changes in the contents of 5-HMF, amino acids, sugars, pH and surface color (Liu et al., 2008, 2009). Among these indicators, polysaccharides were closely correlated to the major biological activities of PMP, and an HPLC-evaporative light scattering detection (ELSD) method was proposed for qualification of the sugars in PMP, with the major constituents assigned as glucose, fructose, and sucrose. The contents of D-fructose and sucrose decreased, while the content of D-glucose increased (Liu et al., 2009). In another report, the content of low molecular weight polysaccharides increased after processing (Qiu et al., 2007).

### Quality control of PMP

PMP is one representative processed drug that needs repeated steaming. According to the record of ancient writings (Cui et al., 2017), nine cycles of steaming and solarization were required, but in current practice, this traditional method was commonly replaced with steaming once within a few hours. Moreover, PM and its adulterants stained with black dye to simulate PMP without processing were found in the market. It was noted that the pharmacopeia protocols failed to differentiate PM from PMP mainly due to the poor specificity, as the same targets including TSG, emodin and physcion were determined in both of these two medicines. All of these factors would pose a serious health risk. Therefore, the critical markers should be screened and identified in order to distinguish PM and PMP as well as evaluate the quality of PMP. On this occasion, comparative studies of chemical constituents were carried out based on the platform of HPLC-DAD and mass spectrometry (Liang et al., 2010; Liu et al., 2011). 5-HMF was first proposed as a key ingredient to authenticate PMP and PM on account of it being recognized as a product of the Maillard reaction and being newly formed during processing (Liu et al., 2009). However, some scientists found contrary results that 5-HMF could not be observed in PMP in their HPLC analyses (Wu et al., 2012). Furthermore, 5-HMF was a controversial agent due to its toxicity (Severin et al., 2010; Bauer-Marinovic et al., 2012; Islam et al., 2014), as studies had shown that 5-HMF exhibited cytotoxic, genotoxic and tumoral effects. On the other hand, six ingredients, namely catechin, flavanol gallate dimer, polygoninmitin B, emodin-1-*O*-glucoside, emodin-8-*O*-(6′-*O*-malonyl)-glucoside, and physcion-8-*O*-(6′-*O*-malonyl)-glucoside, disappeared or decreased significantly after processing, which were assigned as chemical markers for differentiating PM from PMP (Liang et al., 2010; Liu et al., 2011). Nevertheless, the repeatability and reliability of this strategy needs to be validated in further research, which is mainly due to that the amounts of these markers were relatively minor compared to other components in PM before steaming, and the batches of samples investigated in the studies were limited.

As mentioned above, it was difficult to screen the markers only from the perspective of chemical compounds because variations in the plant origins and processing technologies of PM would result in significant differences of index components. In order to assess the quality of PMP, some new approaches were performed based on chemical profiling combined with a bioactivity assay. Chang et al. (2016) conducted an activity-based integrated UHPLC/Q-TOF-MS-FC method to clarify the effect of the processing time on the lipase inhibitory activity of PMP. Chen et al. (2016) developed an online HPLC-DAD-CL assay based on the three reactive oxygen species to evaluate the quality of PMP. In addition, in some other methods, the chemical compositions were analyzed and toxicity monitoring was established to evaluate the processing technologies and quality of PMP (Pang et al., 2014; Ma Z. J. et al., 2015).

## Chemical analysis of hepatotoxic components

### Analysis of the proposed toxic ingredients

Currently, the hepatotoxicity of PM has attracted great concern, and a considerable number of experiments related to PM-induced liver injury were carried out, which offered us comprehensive information to understand the mechanisms. In general, the extracts of PMP are considered to be relatively safe, while hepatotoxicity is found in the PM extracts. Several publications have focused on comparatively studying the toxicities of various extraction solvents from PM and PMP, and the results suggested that the ethanol extract could induce hepatic lesions more easily than that of water decocta (Lv et al., 2013, 2015; Lin et al., 2015e). The order of toxicity was described as PM ethanol extract > PM water extract > PMP ethanol extract > PMP water extract, and in another manuscript (Wu et al., 2012), the toxicity order was proposed as follows: PM water extract > PM acetone extract > PMP acetone extract. Although the hepatotoxic chemicals attributing to the hepatic lesions of PM remain in dispute, emodin and its derivates were believed to be the most likely hepatotoxic components (Yu et al., 2011; Ma J. et al., 2015), and they have also gained much attention from researchers. Recent studies indicated that emodin showed severe cytotoxicity against the human liver cell line L-02 in a concentration- and time-dependent manner. Furthermore, a time-dependent intracellular accumulation of emodin was found in cellular toxicokinetic research by using an HPLC-MS method (Li et al., 2012). Subsequently, an experiment emphasizing the multicomponent interactions of PM was conducted by the same research group, and the results suggested that TSG could delay the elimination of emodin, with the mechanism possibly associated with the inhibition of UGT1A8 mRNA expression (Ma et al., 2013). Lv et al. (2015) explored the toxic components of PM based on biospecific hepatocyte extraction, and the results demonstrated that emodin, physcion, EG and PG were proposed as hepatotoxic components. Lin et al. (2015e) established an UPLC-Q-TOF/MS approach coupled with Progenesis QI and Makerlynx XS software to screen the toxic components from extractions of PM, in which the suspected targets were recognized as emodin-*O*-(malonyl)-hex, emodin-*O*-glc, emodin, emodin-8-*O*-glc, emodin-*O*-(acetyl)-hex, and emodin-*O*-hex-sulfate. It was noteworthy that some reports speculated that the toxicity of PM might not be correlated with the content of emodin derivatives but depended on the contents of TSG or the relative content of TSG and emodin (Wu et al., 2012; Yang M. et al., 2016), which is mainly because the amount of emodin was relatively small in PM. Actually, the idiosyncratic hepatotoxicity induced by the isomerization of TSG (*cis*-TSG) in LPS-treated rats was found in the latest publication (Li et al., 2017), which provided us a new perspective on liver injury by PM. In addition, tannin is another major component in PM, which accounts for approximately 15% of the total dry weight. It was hypothesized that tannin is one of the reasons for the induced liver damage mainly because the content of tannin decreased by 9% after processing (Liu et al., 2005), which meanwhile attenuated the toxicity. In a preliminary investigation, the significant changes of liver biochemical indices were observed after the oral administration of tannin extracts of PM (Hu et al., 2010, 2011). However, in current practice, the chemical analysis approaches regarding tannin are still in the beginning stages, and no individual reference is available due to its complicated structure. Furthermore, the influence of some other factors, i.e., specification (Li Y. M. et al., 2016) and geographical areas (Lin et al., 2017), on the hepatocyte toxicity of PM were discussed. Table 6 summarizes the chromatographic methods for the hepatotoxic analysis of PM *in vitro* and *in vivo*.

**Table 6:** Hepatotoxic analysis of PM *in vivo* and *in vitro*.

**Techniques**	**Analytes**	**Species, administration and biological sample**	**Details**	**References**
HPLC-DAD (290 nm)	TSG, EG, PG, emodin, physcion, chrysophanol	Mice, p.o., repeated 28 days (5, 10 and 20 g/kg/day of water and acetone extracts of PM and PMP, respectively), blood samples, histopathologic examination and biochemical analysis	Eluted with CH_3_CN: H_2_O (0 min: 10:90; 35 min: 40:60; 60 min: 100:0) on a hypersil C_18_ column	Wu et al., 2012
HPLC-MS	TSG, emodin, EG	Rats, p.o., repeated 21 days (1 and 20 g/kg/day of 80% ethanol extractions of PM), blood samples and tissues, histopathologic examination and biochemical analysis	Eluted with CH_3_CN: H_2_O (containing 0.1% HCOOH) (0 min: 20:80; 3.5 min: 35:65; 4.5 min: 35:65; 6 min: 40:60; 7 min: 40:60; 8 min: 100:0; 11 min: 100:0) On an Agilent Extend-C_18_ column	Ma J. et al., 2015
HPLC-MS	TSG, emodin	Rats, p.o. repeated 7 days (TSG, 117 mg/kg), on the 8th day, p.o. (emodin 82.4 mg/kg), blood samples	Eluted with CH_3_CN: H_2_O (containing 0.1% HCOOH) (0 min: 70:30; 2 min:70:30) On an Agilent Extend-C_18_ column	Ma et al., 2013
HPLC-DAD (254 nm)	TSG, emodin, physcion	Human hepatocytes cell L-02, treated with serial concentrations of water, 50% and 95% ethanol extracts of PM and PMP (20~100 μg/mL, respectively), MTT assay	Eluted with CH_3_OH: H_2_O (containing 0.1% H_3_PO_4_) (0 min: 40:60; 5 min: 70:30; 10 min: 80:20; 15min: 85:15; 20min: 90:10; 25 min: 90:10) On a Zorbax SB-C_18_ analytical column.	Yu et al., 2011
HPLC-DAD (210, 280, 320 nm)	15 components	Human hepatocytes cell L-02, treated with serial concentrations of water and ethanol extracts of PM (0, 0.5, 1, 2.5, 5 mg/mL, respectively), MTT assay	Eluted with CH_3_CN: H_2_O (containing 0.1% HCOOH) (0 min: 6:94; 7 min: 6:94; 12 min: 6:94; 20 min: 8:92; 22 min: 12:88; 50 min: 25:75; 55 min: 0:100) on a Zorbax SB-AQ C_18_ column	Lv et al., 2015
UPLC-Q-TOF-MS	Non-targeted	Human hepatocytes cell L-02, treated with serial concentrations of water and ethanol extracts of PM and PMP (7.81~1,000.0 μg/mL, respectively), MTT assay	Eluted with CH_3_CN: H_2_O (both of them containing 0.1% HCOOH) (0 min: 0:100; 3 min: 10:90; 10 min: 20:80; 20 min: 70:30; 21 min: 100:0; 21.1 min: 0:100; 25 min: 0:100) on an ACQUITY UPLC HSS T3 column	Lin et al., 2015e
HPLC-MS	emodin	Human hepatocytes cell L-02, treated with serial concentrations of emodin (0, 10, 20, 40, 60, 120 μM), Cell Counting Kit (CCK)-8 assay	Eluted with CH_3_CN: H_2_O (containing 0.2% HCOOH) (0 min: 45:55; 15 min: 30:70) On a C_18_ column	Li et al., 2012
UPLC-MS	emodin	Human hepatocytes cell L-02, treated with serial concentrations of emodin (10~120 μM), MTT assay	Eluted with CH_3_CN: H_2_O (containing 0.1% HCOOH) (0 min: 5:95; 3 min: 50:50; 15 min: 100:0; 20 min: 100:0; 21 min: 5:95; 26 min: 5:95) On a Zorbax Eclipse plus C_18_ column	Liu et al., 2015

### Identification of the metabolites

Exploration of the metabolites plays an important role in clarifying the possible mechanism associated with the liver damage resulting from PM. Lin et al. (2015c) established an U-HPLC-Q-TOF/MS method to describe the absorption and metabolism of PM extract in rat plasm after oral administration, and 16 of 23 compounds were indicated as prototype components of PM, while seven compounds were predicted to be metabolites, which included three isomers of stilbene glucoside glucuronidation, two isomers of emodin glucuronidation, torachrysone glucuronidation, and torachrysone. Through an *in vitro* study, the metabolism of active compounds of PM was investigated in human normal liver cells (L-02) by means of LC-MS (Liu et al., 2015) and HPLC (Lin et al., 2015), and the results suggested that eight phase II metabolites of TSG and emodin were detected. Their chemical structures were elucidated based on their characteristic fragments, including three isomers of the glucuronidation of TSG, three isomers of the glucuronidation of emodin, one sulfation of emodin, and one emodin-cysteine adduct. In addition, the formation of emodin metabolites mediated by cytochrome P450 was investigated (Qin et al., 2016), and three hydroxylation metabolites named 2-hydroxyemodin, 5-hydroxyemodin, and ω-hydroxyemodin as well as three *N*-acetyl cysteine conjugates and two emodin-derived GSH conjugates were identified. Among these metabolites, emodin-cysteine was suspected to be associated with liver injury due to the formation of an adduct disturbing GSH and fatty acid metabolism in human liver cells. However, further validation should be carried out to confirm this hypothesis. Due to the multiple phenolic hydroxyl groups in TSG and emodin as well as the limitation of the mass spectrum, the combined positions of glucuronide/ sulfatide/cysteine were still uncertain. In addition, the bioactivities and toxicities of these metabolites need to be evaluated.

### Metabolomics studies

In last few years, studies used metabolomics methods integrated with pattern recognition to investigate the potential hepatotoxicity of PM have been constantly reported, which provided preliminary information on the mechanisms of liver injury induced by PM (Dong et al., 2015; Zhang et al., 2015; Li et al., 2016; Zhang C. E. et al., 2016; Ma et al., 2017; Xia et al., 2017). It should be noted that in these experiments, the rats were usually administrated a high dose of PM orally for more than 28 days. In a targeted metabolomics study, the perturbation of nine bile acids (BAs) associated with PM-induced liver injury were evaluated. The glycodeoxycholic acid (GDCA) in bile and hyodeoxycholic acid (HDCA) in serum significantly decreased and were assigned as potential biomarkers for PM-induced liver injury in rats (Dong et al., 2015). In untargeted metabolomics research, 16 possible endogenous metabolites in serum along with 10 metabolites in liver tissue samples were identified by means of GC-MS, and these markers were involved in amino acid, fatty acid, and energy metabolism pathways (Zhang et al., 2015; Xia et al., 2017). Additionally, 16 significantly disturbed biomarkers in urine samples were authenticated by the LC-MS assay, and the pathway analysis showed that vitamin B6 metabolism, tryptophan metabolism and the citrate cycle might be the most important pathways involved in the PM-induced hepatotoxicity (Zhang C. E. et al., 2016). Furthermore, 21 potential metabolomics biomarkers related to the idiosyncratic hepatotoxicity of PM were detected by an UHPLC-MS approach, which is mainly associated with the tricarboxylic acid cycle and sphingolipid metabolism pathways (Li et al., 2016). Table 7 summarizes the chromatographic methods for the metabolomics analysis of PM.

**Table 7:** Metabolomics analysis of PM.

**Techniques**	**Biomarkers**	**Pathway analysis**	**Species, administration, and biological sample**	**Details**	**References**
LC-MS	GDCA, HDCA	bile acids metabolism	Rats, p.o., repeated 42 days (50 g/kg/day of 75% ethanol extracts of PM and PMP, respectively), blood and bile samples	Eluted with CH_3_OH (containing 0.1% HCOOH): H_2_O (containing 0.1% HCOOH and 1mM CH_3_COONH_4_) (0 min: 70:30; 3 min: 80:20; 8 min: 90:10; 8.5 min: 95:5; 14.4 min: 100:0; 14.5 min: 70:30; 20 min: 70:30) On a Ulimate C_18_ column	Dong et al., 2015
GC-MS	18 biomarkers	amino acid, lipid and energy metabolism	Rats, p.o., repeated 28 days (30 g/kg/day of water and 80% ethanol extracts of PM, respectively), blood and liver tissues	The temperature program was 0–5 min: 80°C, 6–23 min: 80–260°C, 24–34 min: 260°C, on a HP-5MS column	Zhang et al., 2015
GC-MS	10 biomarkers	amino acid, fatty acid, and energy metabolism	Rats, p.o., repeated 28 days (19.2, 192, and 1920 mg/kg/day of 95% ethanol extracts of PM), serum samples	The temperature program was 0–25 min: 80–280°C, 25–29 min: 280°C, on a DB-5MS column	Xia et al., 2017
LC-MS	16 biomarkers	vitamin and tryptophan metabolism, citrate cycle	Rats, p.o., repeated 28 days (20 g/kg/day of 75% ethanol extractions of PM and PMP, respectively), urine samples	Eluted with CH_3_OH : H_2_O (0 min: 30:70; 5 min: 90:10; 5.1 min: 90:10; 40 min: 30:70) On an Agilent ZORBAX SB-C_18_ column	Zhang C. E. et al., 2016
UHPLC-MS	21 biomarkers	sphingolipid metabolim and tricarboxylic acid cycle	Rats. p.o., different extracts of PM, blood samples	Eluted with H_2_O: CH_3_CN (both of them containing 0.1% HCOOH(0 min: 95:5; 1 min: 95:5; 9 min: 60:40; 19 min: 10:90; 21 min: 0:100; 25min: 0:100) On a ZORBOX RRHD C_18_ analytical column	Li et al., 2016

### Exogenous contaminants

Exogenous contaminants (e.g., mycotoxins, heavy metals, and pesticides) are also considered to be the main reasons for the cause of herbal (Drug-Induced Liver Injury) DILI, especially, the process of steaming would make aflatoxins easily appear if the solarization of PMP was not in time, which usually is known as its acute hepatotoxicity and carcinogenic feature. Several publications focused on the determination of these mycotoxins by means of UHPLC-MS, including aflatoxins B1, B2, G1, G2, M1, and M2 (Han et al., 2010a), ochratoxins A and B (Han et al., 2010b), fumonisins B1, B2, and B3 (Han et al., 2010c), five type B trichothecenes, which contained deoxynivalenol (DON), 3-acetyldeoxynivalenol (3-ADON), 15-ADON, nivalenol and fusarenon X (Han et al., 2010d), zearalenone (ZEN) and its derivatives (Han et al., 2011). It should be noted that the isotope dilution method was employed in all of the experiments mentioned above, which is attributed to the advantage that the isotopic IS had similar behavior to the target during the sample pretreatment and ionization process. Thus, in this way the matrix effects were minimized, and the recoveries were calibrated. The result revealed positive findings of ZEN (1.1 μg/kg) as well as fumonisins B1 (1.25 μg/kg) and B2 (0.82 μg/kg) in randomly selected PM samples, respectively. On the other hand, the heavy metals and inorganic elements were evaluated by using inductively coupled plasma mass spectrometry (ICP-MS) (Luo et al., 2014, 2015f,g) and atomic absorption spectrometry (AAS) (Shi et al., 2011), and the results showed that parts of samples with detected Hg, As and Pb exceeded the safety limits specified by the Green Trade Standards of Importing and Exporting Medicinal Plants and Preparations of China (Pb ≦ 5 μg/g, Cd ≦ 0.3 μg/g, Hg ≦ 0.2 μg/g, Cu ≦ 20 μg/g, and As ≦ 2 μg/g), which should raise significant concerns regarding this issue. In another translational medicine study, five batches of identified PM or PMP were collected from patients with suspected PM DILI, and hazardous materials of these samples, which are comprised of heavy metals, mycotoxins, and pesticides, were determined according to the Chinese pharmacopeia or European Union standards. The laboratory reports demonstrated that there were no targets exceeding the safety limits (Wang J. et al., 2015).

## Conclusions and future perspectives

As one of the most widely used traditional medicines in China, PM and its processed products have been widely used for the clinical treatment of fatty liver disease, hyperlipidemia, cirrhosis, hepatitis B, learning and memory obstructions, Alzheimer's disease and Parkinson's disease (Lin et al., 2015a; Li et al., 2016a; Ling and Xu, 2016). In recent years, the hepatotoxicity of PM has been well-documented, but the mechanisms of the toxicity remain unknown. Moreover, the quality evaluation of PMP has attracted great concern due to that the processing procedure could significantly decrease the toxicity. However, the processing mechanism was still unclear, and a scientific quality standard to control the quality of PMP was lacking. In the current review, we summarize the existing studies on the chemical analysis of PM and PMP, and a considerable amount of experimental works were carried out that focused on the difficult points mentioned above. Nevertheless, the following aspects still require investigation.

First, systemic chemical constituent studies could lay the foundation for the deeper understanding of the pharmacological efficacies, adverse effects, qualitative determination as well as quantitative analysis of PM and PMP. Besides Lin et al. (2015a) summarized 103 chemical compounds of PM, one new type agents were discovered by our group (Yang J. B. et al., 2016; Yang et al., 2017a,b), more than 30 novel dianthrone glycosides were elucidated unambiguously by spectroscopic analysis. In addition, the toxicities of parts compounds were evaluated against L-02 cell lines and KB tumor cell lines, and the results indicated that these constituents showed moderate hepatotoxicities. These findings provided us a new perspective on liver injury by PM. Apart from these dianthrone glycosides, novel dimeric stilbene glucosides along with polysaccharides were found in the last 2 years (Yan et al., 2014; Park et al., 2016; Zhang and Cui, 2016; Zhao et al., 2016; Zhu et al., 2016, 2017), and their anti-neuroinflammatory effects, antioxidant and antitumor properties were evaluated. In this way, careful chemical exploration should be performed involving the conventional phytochemistry methods as well as MS techniques which depend on the standards and fragmentation pattern rules of references.

Second, the detailed transformation of major compounds in PM during the processing procedure could allow us to better understand the mechanisms of preparation, which would facilitate the establishment of a quality control method and the normalization of the processing technology. As we discussed in this review, combined anthraquinones were unequivocally hydrolyzed into free ones, but the compounds from the degradation of TSG were difficult to detect. Additionally, the concentration of TSG decreased drastically after processing, and the most predominant constituents in PM-TSG seemed to disappear. In fact, the basic TSG structure that consists of two aromatic rings, which are linked through one alkene double bond, is a focus of reactivity. TSG was speculated to be easily degraded or isomerized under different conditions (Figueiras et al., 2011). Several studies focusing on the stability of TSG have been carried out, and the results demonstrated that the degradation of TSG was pH-, temperature-, irradiation- and metal ion-dependent. Two degradants together with one isomerized product were observed in acidic, alkaline and irradiation conditions (Sun et al., 2009b; Ren et al., 2011; Wang et al., 2011), respectively. In addition, the products of the TSG dimer with a water molecule were found in water containing Fe^3+^ (Li R. Y. et al., 2016) and with H_2_O_2_ (Lv et al., 2008) (Supplementary Figure S3). It is hypothesized that there are two pathways associated with the transformation mechanisms of TSG. On the one hand, due to the alkene double bond is a focus reactivity of TSG, the degradation reaction was occurred, and the transformation products might be the small molecules as phenolic acids (Lv et al., 2008; Li R. Y. et al., 2016). On the other hand, the polymerisation was happened, the transformation products might be the dimers of TSG, and this speculation is based on the results of TSG in Fe^3+^ solutions (Li R. Y. et al., 2016) or H_2_O_2_ (Lv et al., 2008), but also the stilbene glucoside dimers were isolated from processed roots of *P. multiflorum* (Yan et al., 2014). Furthermore, concerning the transformation products of TSG were newly formed after preparation and also their contents changed with the variation of processing time, these targets were proposed as the critical markers in assessing the quality of *P. multiflorum* praeparata.

## Author contributions

SM and SL conceived the review; YL wrote the manuscript; XG, JY, and WL collected the literatures; and QW edited the manuscript. All the authors read and approved the final version of the manuscript.

### Conflict of interest statement

The authors declare that the research was conducted in the absence of any commercial or financial relationships that could be construed as a potential conflict of interest.
