# Supplementary material for: Polygonum multiflorum Thunb.: A Review on Chemical Analysis, Processing Mechanism, Quality Evaluation, and Hepatotoxicity
Source: Front Pharmacol. 2018 Apr 16;9:364. doi: 10.3389/fphar.2018.00364 (PMC5912012; doi:10.3389/fphar.2018.00364)
Supplement: Supplementary file 1 [file Presentation1.pdf]

Supplementary Material

Polygonum multiflorum Thunb.: A Review on Chemical Analysis, Processing Mechanism, Quality Evaluation and Hepatotoxicity

Yue Liu[1,2], Qi Wang[2], JianBo Yang[2], XiaoHan Guo[2], WenXi Liu[2], ShuangCheng Ma[2,3*], ShaoPing Li[4*]

*** Correspondence:**
ShuangCheng Ma, E-mail: masc@nifdc.org.cn.
ShaoPing Li, E-mail: spli@umac.mo, lishaoping@hotmail.com.

Supplementary Figures

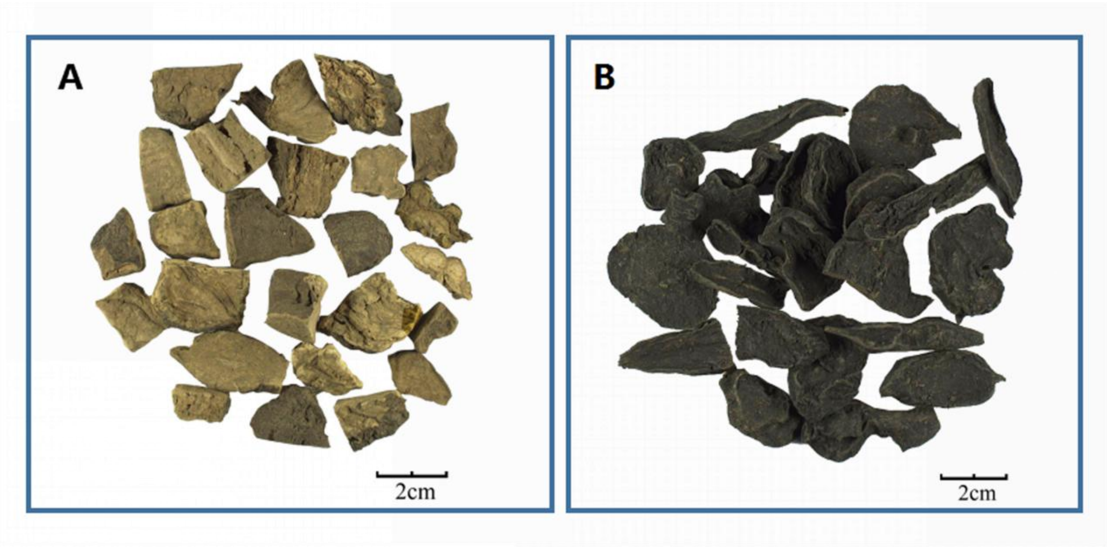

Supplementary Figure 1. The roots of *Polygonum multiflorum* **(A)**; the roots of *P. multiflorum* Praeparata **(B)**.
Note: Based on the botanical perspective, *Polygonum multiflorum* Thunb. is a synonym of *Reynoutria multiflora* (Thunb.) Moldenke.

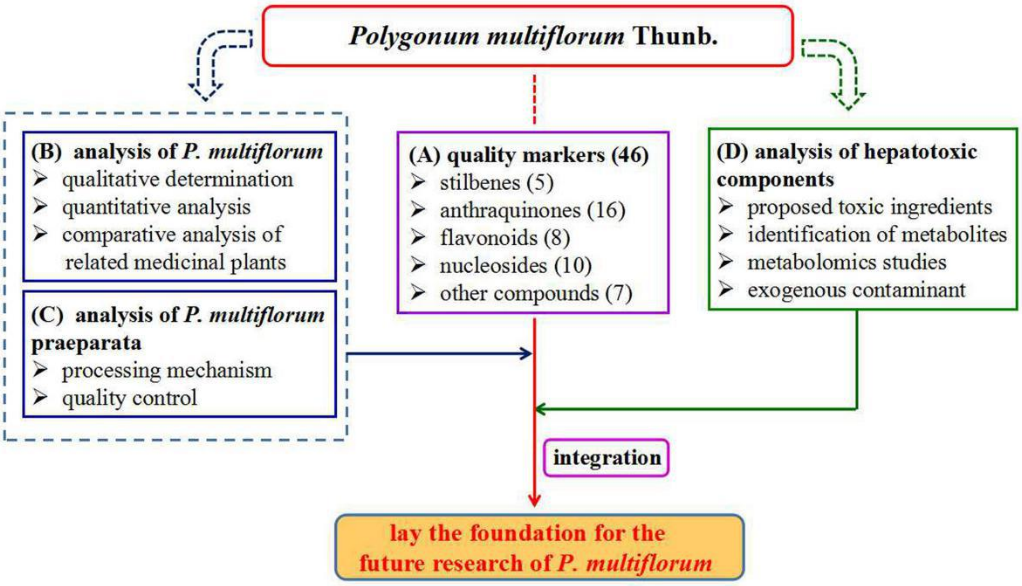

Supplementary Figure 2. Schematic diagram of the review process.

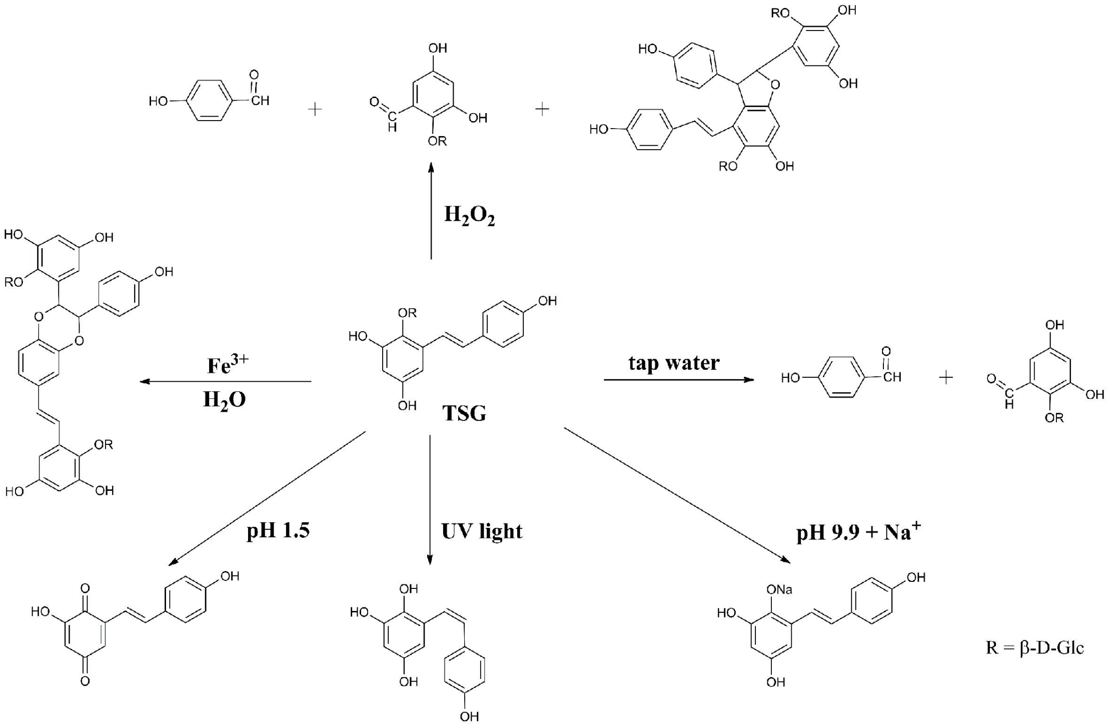

Supplementary Figure 3. Proposed transformation pathway of TSG in different solutions.
